# A CRISPR screen identifies MAPK7 as a target for combination with MEK inhibition in *KRAS* mutant NSCLC

**DOI:** 10.1371/journal.pone.0199264

**Published:** 2018-06-18

**Authors:** Nicholas Dompe, Christiaan Klijn, Sara A. Watson, Katherine Leng, Jenna Port, Trinna Cuellar, Colin Watanabe, Benjamin Haley, Richard Neve, Marie Evangelista, David Stokoe

**Affiliations:** 1 Department of Discovery Oncology, Genentech Inc., South San Francisco, CA, United States of America; 2 Department of Bioinformatics, Genentech Inc., South San Francisco, CA, United States of America; 3 Department of Molecular Biology, Genentech Inc., South San Francisco, CA, United States of America; University of South Alabama Mitchell Cancer Institute, UNITED STATES

## Abstract

Mutant *KRAS* represents one of the most frequently observed oncogenes in NSCLC, yet no therapies are approved for tumors that express activated KRAS variants. While there is strong rationale for the use of MEK inhibitors to treat tumors with activated RAS/MAPK signaling, these have proven ineffective clinically. We therefore implemented a CRISPR screening approach to identify novel agents to sensitize *KRAS* mutant NSCLC cells to MEK inhibitor treatment. This approach identified multiple components of the canonical RAS/MAPK pathway consistent with previous studies. In addition, we identified *MAPK7* as a novel, strong hit and validated this finding using multiple orthogonal approaches including knockdown and pharmacological inhibition. We show that MAPK7 inhibition attenuates the re-activation of MAPK signaling occurring following long-term MEK inhibition, thereby illustrating that MAPK7 mediates pathway reactivation in the face of MEK inhibition. Finally, genetic knockdown of MAPK7 combined with the MEK inhibitor cobimetinib in a mutant *KRAS* NSCLC xenograft model to mediate improved tumor growth inhibition. These data highlight that MAPK7 represents a promising target for combination treatment with MEK inhibition in *KRAS* mutant NSCLC.

## Introduction

The RAS/MAPK signaling pathway plays a critical role in embryogenesis, tissue growth and repair, and normal tissue homeostasis downstream of growth factor activation. Active RAS signals through the RAF kinases that in turn activate a signaling cascade through the MEK and ERK kinases, resulting in the phosphorylation of numerous effector proteins to promote appropriate cellular programs. Consistent with this role in normal tissue homeostasis, activation of the RAS/MAPK pathway plays a prominent role during oncogenic transformation, tumor growth and maintenance [1]. Widespread mutation and/or amplification of various genes (*KRAS*, *HRAS*, *NRAS*, *BRAF*) comprising the components of MAPK pathway or its upstream activators (RTKs, such as EGFR, MET) are correlated with constitutive activation of the pathway in a significant proportion of human cancers, including melanomas, non-small cell lung (NSCL), colorectal, and pancreatic cancers [2]. Accordingly, there has been significant effort directed towards developing inhibitors against various components of this pathway [3]. Some of these efforts have shown substantial benefit, such as the development of EGFR inhibitors for the treatment of *EGFR* mutant NSCLC. The on-target nature of this effect is demonstrated by the fact that second site mutations in *EGFR* are frequently found upon progression that reduce drug effectiveness [4, 5]. Similarly, BRAF inhibitors result in initial dramatic responses in *BRAF* mutant melanoma and NSCLC, however these tumors also progress on therapy through additional acquired genetic alterations in *BRAF* and other factors resulting in pathway reactivation [6]. Addition of MEK inhibitors to BRAF inhibitors has extended the response of *BRAF* mutant tumors to therapy, illustrating the utility of targeting downstream kinase activation in this context [7]. Despite these successes and the multiple tumor-associated mutations resulting in activation of MEK, clinical benefit to MEK inhibitors has been relatively modest, particularly in the context of tumors harboring mutant *RAS* [8]. To identify additional druggable proteins and pathways that mediate pathway reactivation following MEK inhibition we performed a CRISPR screen in *KRAS* mutant NSCLC cell lines. Following extensive validation of hits from this screen we identified MAPK7, also known as ERK5, as a factor that mediates pathway reactivation following MEK inhibition, thereby identifying MAPK7 as a promising target for combination with MEK inhibitors.

## Materials and methods

### Cell lines, and inhibitors

All cell lines were obtained from the Genentech cell bank, gCELL. The cell lines used for this study included were MOR (ECACC), NCI-H2122 (ATCC), A549 (ATCC), NCI-H441 (ATCC). Small Molecule inhibitors were either purchased from outside vendors or generated at Genentech. S1 Table lists the inhibitor name, expected target, source and relevant catalogue number.

### Culture methods

Cell lines above were all maintained in RPMI-1640 (Gibco), with 10% FBS (Sigma) and 2 mM L-glutamine. Upon introduction of Cas9 into each cell line they were subsequently maintained in culture media with 10 μg/mL of Blasticidin.

### Viral constructs

*Cas9* was cloned into the pLENTI6.3 vector (ThermoFisher #V53306) which contains a Blasticidin selection marker. The gRNA vectors are based on Sigma's pLKO1.5 lentiviral vector (product #SHC-201). The gRNA construct library (based on sequences designed at Genentech) and subsequent virus utilized for this study were generated by Cellecta, Inc. Inducible shRNA sequences used in this study were designed and generated at Genentech and introduced into cells using the pINDUCER10 lentiviral vectors [9].

### CRISPR library screen

The *KRAS* mutant lung cell line MOR was stably transduced with *Cas9*. Expression and function of Cas9 was confirmed by western blot and knockdown of both CD81 and PLK1. We performed three independent infections with virus generated from a ‘druggable genome’ gRNA library (2194 genes, 8 gRNAs/gene, S2 Table) at a MOI ~0.3 at 1000X coverage to establish three replicate populations. Briefly, 60 million cells were infected with the lentiviral based “druggable genome” gRNA library S2 Table) in 6-well format, immediately spun at 2000 rpm for 2 hrs in the presence of 8 μg/mL polybrene. 48 hrs after infection three stable populations were established by passage into 3 μg/mL puromycin. After seven days cells were split into three treatment arms: DMSO, MEKi (0.5 μM cobimetinib), and ERKi (1 μM GDC-0994). Each arm was seeded with a minimum of 20 million cells to maintain 1000x representation of each gRNA in the library. The remaining cells (> 20 million cells) were retained as a reference genomic sample (D0). Cells were maintained in media containing the indicated treatments and were split approximately every 3 days (~80–90% confluence). At each split a new flask was seeded with 20 million cells and the remaining cells were retained for the harvest of genomic DNA. A small sub-library of CRISPR gRNA constructs encompassing hits from the first screen (targets described in S3 Table) was generated. Virus was generated and infected into MOR, A549, NCI-H441, and NCI-H2122 cell lines stably expressing Cas9, in the manner described for the ‘druggable genome’ gRNA library screen. Stable populations of transduced cells were treated as described above.

### Genomic DNA prep and NGS sequencing

Cell pellets were lysed and prepped using Qiagen Genomic DNeasy kit. PCR reactions were performed to amplify the gRNA sequence from 130 μg of genomic DNA from each sample, using high fidelity amplification kit (Thermo F530L). Subsequent library prep for sequencing was performed using the Nugen Ovation Library System for Low Complexity samples. Paired End Sequencing of the CRISPR screen libraries was carried out in Hiseq 2500 in Rapid mode—2x150 cycles. Reads were aligned to guide sequences using the GSNAP [10] aligner. For analysis guides with less than 10 reads over all samples were discarded. To normalize libraries for total read count we used the edgeR package [11] and to calculate significant differential guide abundances at the 21 day time point between treatment and control arms we used the voom/limma package [12], both in the R programming language.

### Array gRNA validation

A smaller library (S4 Table) was used to validate gRNAs. These gRNAs were used to generate virus in a 96-well plate (100 μL). Virus (5 μL) was used to infect quadruplicate 96-well plates of A549 cells followed by selection using puromycin (3 μg/mL) for 3 days. After selection, two plates of cells were treated with either cobimetinib (at 50 nM) or DMSO and were allowed to grow until the non-target control (NTC) wells achieved ~90% confluence (3 days). One of these plates from each treatment condition was trypsinized and re-plated into four daughter plates with continued treatment. The other plate was evaluated for cell viability using CellTiter-Glo® (Promega). This process was repeated through four successive passages.

### Interaction between MEK inhibition and test gene depletion

We calculated the difference between predicted inhibition and observed inhibition in the following manner: We calculated the fraction inhibition using f = counts^control^/counts^treatment^. Using this we obtained both the single effects from the guide as well as the drug and their combination. We then calculated the predicted fraction inhibition using f^combo_predicted^ = f^guide^*f^drug^. The difference between observed and predicted was calculated as f^combo_observed^-f^combo_predicted^.

### siRNA validation

MOR, A549, and NCI-H441 were reverse transfected using Lipofectamine RNAiMAX and 100 nM Dharmacon ON-TARGETplus (OTP) siRNA pools directed against *KRAS* (L-005069), *RAF1* (L-003601), *BRAF* (L-003460), *MAPK7* (L-003513), *PAK2* (L-003597), *MCL1* (L-004501), *MARCH5* (L-007001), *MAP2K7* (L-004016) and *DUSP4* (L-003963). Cobimetinib (0.25 μM) or DMSO was added to the cells 48 hrs after transfection. Cells were allowed to grow for 7 days and were evaluated for viability using CellTiter-Glo® (Promega).

### Small molecule inhibitor growth assays

MOR, A549, NCI-H441, and NCI-H2122 were plated in both 384- and 96-well plates at cell densities that would yield ~80% confluence at 96 hrs. Drug was added as a log2 dilution series using a Tecan D300 dispenser 24 hrs after initial cell plating into wells containing either 0.25 μM cobimetinib or DMSO. Cell viability was evaluated using CellTiter-Glo® (Promega) after 72 hrs of additional growth. Dose response curves were generated in Prism7 (Graphpad Software).

### Small molecule inhibitor long-term growth assay

MOR, A549, NCI-H441, and NCI-H2122 were plated at 12,000–50,000 cells per well in 24-well plates. 24 hrs after cell plating drug was added at a two-fold dilution series into wells containing either 0.25 μM cobimetinib or DMSO. Treatment media was replaced every three to four days. After 10 days media was removed and wells were stained with 0.5% crystal violet in 25% methanol for 10 minutes. The plates were imaged using an Oxford Optronix GelCount.

### Western blot analysis

Media was removed and cells washed twice with PBS before being lysed on plate in Peirce/Thermo RIPA (cat#89900) or IP lysis buffer (cat# 87707). Quantification of total protein was performed on sheared/cleared lysate using a bicinchoninic acid (BCA) kit (Pierce/Thermo). 10 μg of protein were separated in 4–12% BIS-TRIS gels and transferred to nitrocellulose membranes via iBlot (Invitrogen). These antibodies were used at 1:1000 with a 5% BSA block: tMAPK7 (CST# 12950), pERK1/2 (CST#4695), pMEK1/2 (CST #9154) and at 1:4000 with 5% BSA block: ßactin (sigma# A2228) for western blot. Protein was visualized using chemiluminescence.

### gRNA and shRNA long-term growth assay with small molecule inhibitors

MOR, A549, NCI-H441, and NCI-H2122 cells were stably infected with one of two gRNA sequences targeting *MAPK7*; gMAPK7_1 (MAPK7_d) 5’-TCCTGTACCAACTGCTGCG-3’ and gMAPK7_2 (MAPK7_e) 5’-GGCCTGAAGTACATGCAC-3’. These cell lines were then plated at low cell density in 12-well plates (between 1,200 and 9,000 cells). Media containing cobimetinib was added 24hrs after initial plating. For evaluation of the effect of shRNA knockdown NCI-H441 and NCI-H2122 cells were stably infected with our top performing shRNA sequence targeting *ERK5*, shRNA sequence: 5’-TAGCGCACGTGTTCCAGTGTG-3’ in pINDUCER10. These cell lines were then plated at low cell density in the 12-well plates (5,000 NCI-H441 cells or 9,000 NCI-H2122 cells) in media containing 250ng/mL of doxycycline. 48hrs after initial plating, media was replaced with media containing cobimetinib. As above, treatment media was replaced every three to four days and after 10 days the media was removed, wells were stained with a crystal violet solution, and plates were imaged using an Oxford Optronix GelCount.

### *In vivo* xenograft tumor studies

NCI-H2122 cells were injected subcutaneously into the left side flank of *NCr nu/nu* mice at 5 x 10^7^ cells per mouse. Xenograft tumors were monitored and measured via caliper. When tumors reached approximately 250 mm^3^, mice were grouped to ensure equivalent tumor volume ranges for each group and treatment was initiated. Cobimetinib was formulated in methylcellulose tween (MCT) at 5 mg/kg, and was administered orally (P.O.) on a daily basis for the duration of the study. Induction of shRNA expression by doxycycline was provided in drinking water at 1 mg/mL in 5% sucrose, ad libitum for the duration of the study. For each study vehicle-treated mice were measured and/or collected. At end of study xenograft tumors were collected and flash frozen in liquid nitrogen and stored at -80°C until processed for further analysis. All animal activities and procedures were performed in accordance with the protocols approved by the Institutional Animal Care and Use Committee (IACUC) for ethical review of animal care and use. All individuals participating in animal care and use are required to undergo training by the institution's veterinary staff. Any procedures, including handling, dosing, and sample collection mandates training and validation of proficiency under the direction of the veterinary staff prior to performing procedures in experimental in-vivo studies.

## Results

### CRISPR knockout screen to identify sensitizers to MEK inhibition in *KRAS* mutant cells

**Fig 1:**
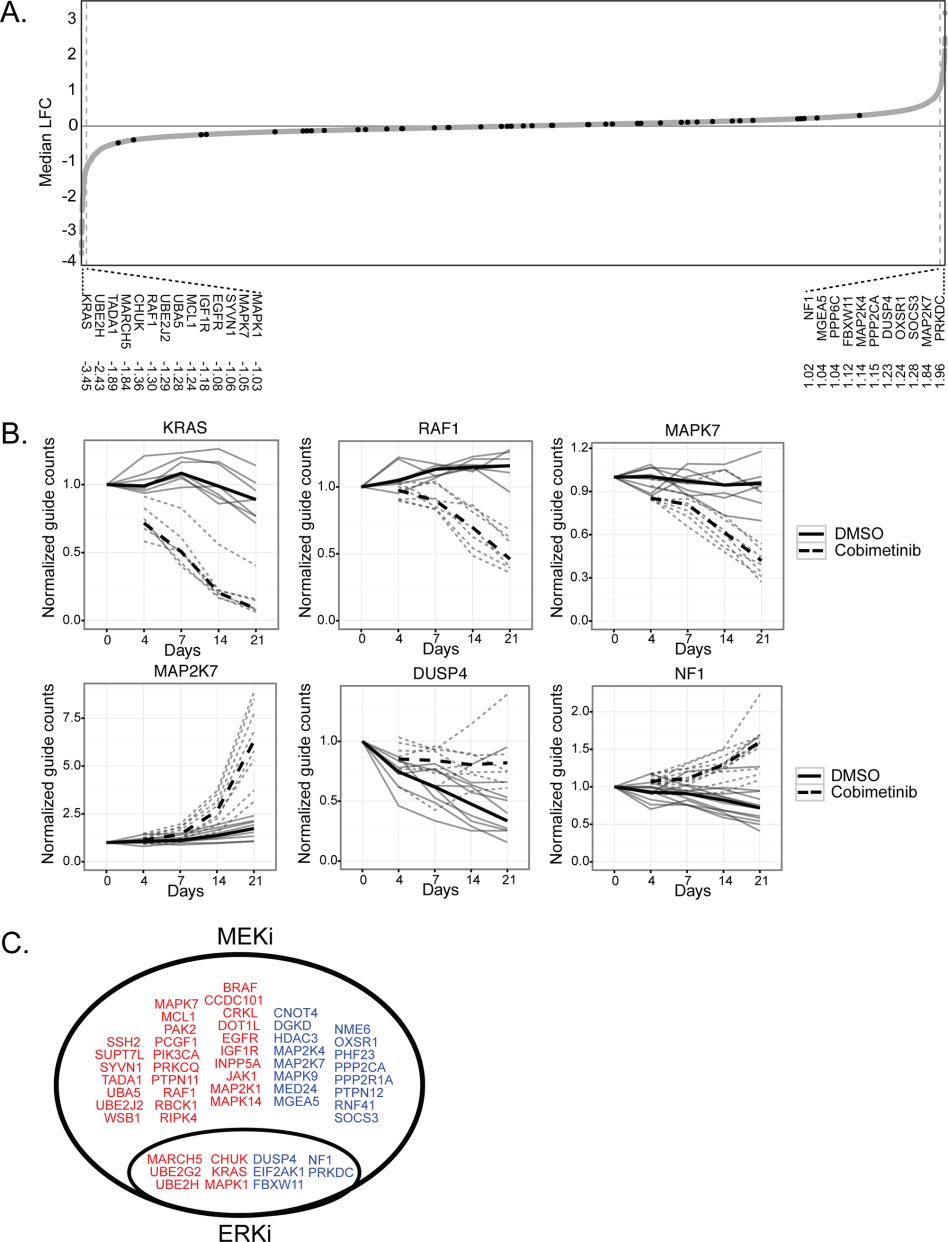
A small set of genes lead to changes in MAPK pathway inhibitor sensitivity. **(A)** Waterfall plot showing the median log fold-change (LFC) in gRNAs targeting each of the ~2200 genes in the library in response to cobimetinib. NTC gRNAs indicated in bold, enlarged are the genes with >1 LFC either in abundance or depletion. **(B)** Examples of the performance of individual gRNAs for genes both enriched and depleted from the pool. Individual gRNAs in the presence of DMSO in thin solid lines, dashed lines in the presence of cobimetinib, thick lines represent the median values. **(C)** Using a >0.5 Median LFC cutoff this Venn-diagram summarizes the small set of genes whose abundance change (depletion in red, enrichment in blue) occurred in both inhibitor arms of the screen. A much larger set of genes showed changes only in the MEK inhibitor arm.

We sought to expand our understanding of the pathways responsible for intrinsic resistance to MAPK pathway inhibition in a *KRAS* mutant lung cancer cell line using a CRISPR knockout sensitizer screen. We chose cobimetinib [13] and GDC-0994 [14] as representative MEK and ERK inhibitors. We selected the MOR *KRAS* mutant NSCLC cell line because its growth rate is relatively insensitive to MEK or ERK inhibitor treatment (S1A Fig) even at concentrations that show significant pathway inhibition (S1B Fig). Consistent with previous reports, resistance to MEK inhibition is characterized by transient pathway inhibition followed by a ‘rebound’ in pathway signaling, contributing to intrinsic drug resistance (S1B Fig) [15, 16]. To identify genetic modifiers of MEK or ERK inhibition, a custom CRISPR lentiviral library targeting kinases, phosphatases, and additional druggable target genes (8 gRNAs/gene, see S1 Table) was transduced into MOR cells stably expressing Cas9. Following puromycin selection, MEK or ERK inhibitors were added to the growth media and cells were collected for gRNA analysis by NextGen sequencing after 4, 7, 14 and 21 days (S1C Fig). The vast majority of the approximately 2000 genes targeted in the CRISPR library, as well as the 40 non-targeting gRNAs, showed very little depletion or enrichment at the 21-day time point of cobimetinib treatment as compared to vehicle treated cells (Fig 1A). However, gRNAs targeting 11 genes showed >2-fold median enrichment, and gRNAs targeting 14 genes showed >2-fold median depletion (Fig 1A). Representative examples of individual gRNAs against three of the top enriched and depleted genes over the time-course of the treatment are shown in Fig 1B. Most of the identified depleted genes encode for components of the MAPK pathway, including *KRAS*, *RAF1* (encoding CRAF) and *MAPK1* (encoding ERK2), and have previously been implicated in sensitizing *KRAS* mutant cell lines to MAPK pathway inhibition. Analysis of the screen performed with the ERK inhibitor GDC-0994 resulted in a subset of the same genes being identified, however no genes unique to the ERK inhibitor treatment were identified, consistent with ERK acting as a dedicated effector of MEK (Fig 1C). Therefore, we focused our attention on the genes identified as modifiers of MEK inhibition.

### Validation of screening hits using knockout and knockdown approaches

**Fig 2:**
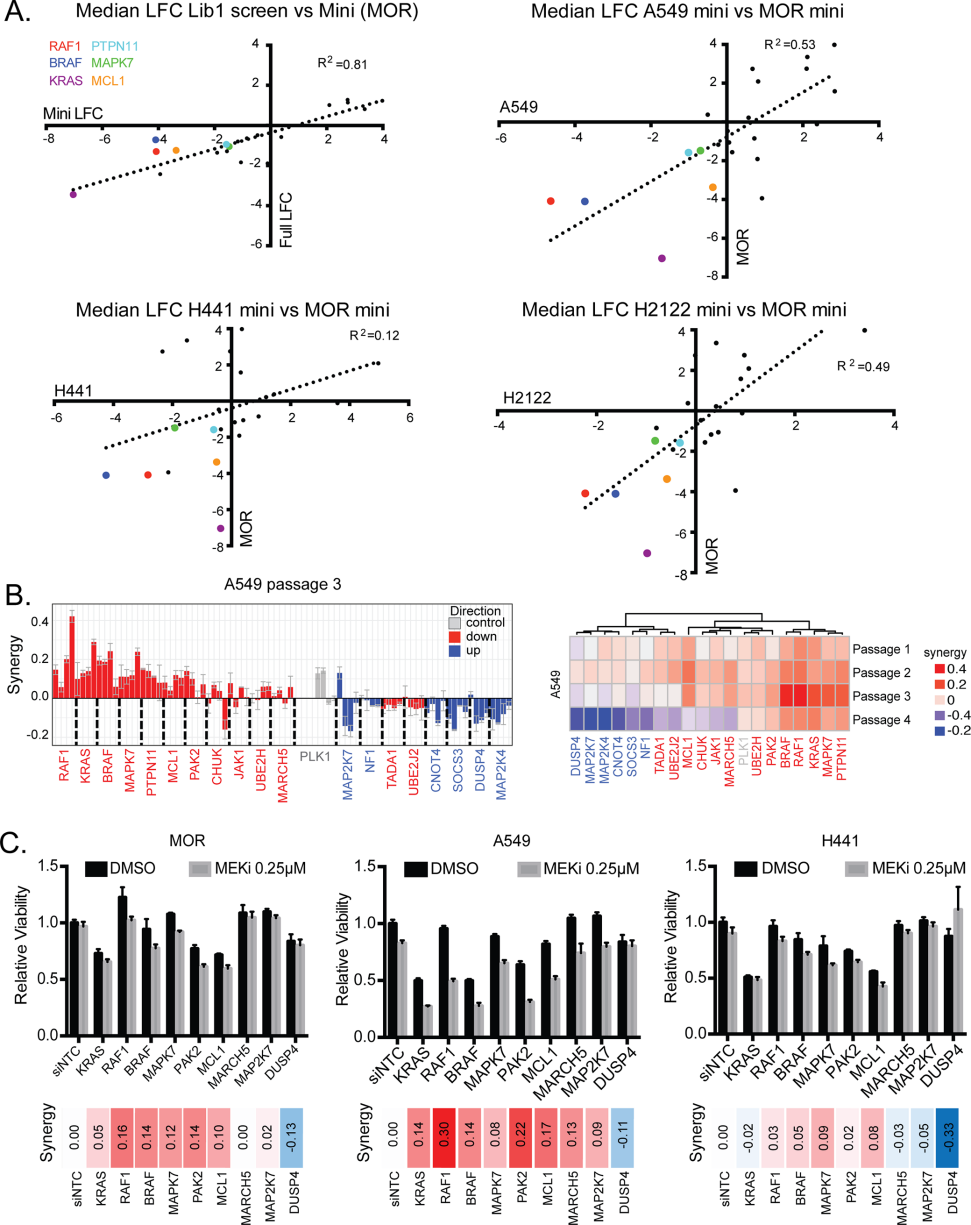
Validation of genes regulating MAPK inhibitor sensitivity in multiple cell lines. **(A)** Four cell lines were subjected to a sub-library pooled screen (mini-pool). The performance of gRNAs in the MOR cell line is compared to activity observed in the original screen. The additional cell lines were then compared to the “mini-pool” performance of the MOR cell line. **(B)** Bar plot showing the combination performance of each of the guides in the sub-library in an arrayed format, at passage 3 in A549 cells. A heat map shows the average combination score across the four guides targeting each gene, across the four passages of the array screen. **(C)** Top: Bar plots showing sensitivity changes in the presence of MEKi with siRNA targeting several hits from the screen. Bottom: combination scores for the siRNA assay.

While several of the genes enriched or depleted in our screen had previously been implicated in influencing response to inhibition of this pathway [15–17], we sought to validate both known and unknown hits in an unbiased manner. We employed a tiered strategy to both reconfirm primary screen in hits in the same assay, as well as identify robust hits that survive several orthogonal validation approaches. For reconfirmation of screen hits, as well as extending the analysis to additional *KRAS* mutant cell lines, we generated a pooled library containing the top four performing gRNAs targeting a set of 21 genes, 15 of which were depleted and 6 of which were enriched in the original pool screen (S3 Table). We then subjected three additional *KRAS* mutant lung cell lines (A549, NCI-H441, and NCI-H2122) in addition to the original screen cell line (MOR) to treatment with cobimetinib over four time points and evaluated the changes in the abundance of the gRNAs by NextGen sequencing. In the MOR cell line, the gRNA abundances showed a high degree of concordance (R^2^ = 0.81) with those of the original screen (Fig 2A). However, the overall correlation between the MOR cell line mini-screen with the other three cell lines was lower (Fig 2A), demonstrating that synthetic lethality of targeting these genes with MEK inhibitors is context dependent. Nevertheless, several genes (*RAF1*, *BRAF*, *KRAS*, *PTPN11*, *MAPK7* and *MCL1*) were identified as being depleted across all tested cell lines in combination with cobimetinib (Fig 2A). To complement the pooled competitive growth assay we also evaluated the performance of each individual gRNA using an arrayed approach. We individually infected cells with each gRNA from 13 of the depleted and 6 of the enriched genes into A549 cells and evaluated the effect on cell growth over multiple passages in the presence or absence of cobimetinib (S2 Fig). We calculated a combination score (difference between expected and observed–described in Materials and Methods) and found that at the 12-day time point (passage 3), most of the depleted gRNAs showed a positive combination score, and most of the enriched gRNAs showed a negative combination score, as expected (Fig 2B). Clustering genes by their combination behavior in this assay clearly identified those with the strongest positive interaction with cobimetinib (*PTPN11*, *MAPK7*, *KRAS*, *RAF1*, *BRAF*) or the strongest negative interaction (*DUSP4*, *MAP2K7*, *MAP2K4*, *CNOT4*). We then utilized siRNA as an orthogonal validation approach, which demonstrated a weaker correlation with the primary screening hits. However some of the more robust hits, such as *RAF1*, *BRAF* and *MAPK7* were confirmed using this method (Fig 2C).

### Validation of screening hits using small molecule inhibitors

**Fig 3:**
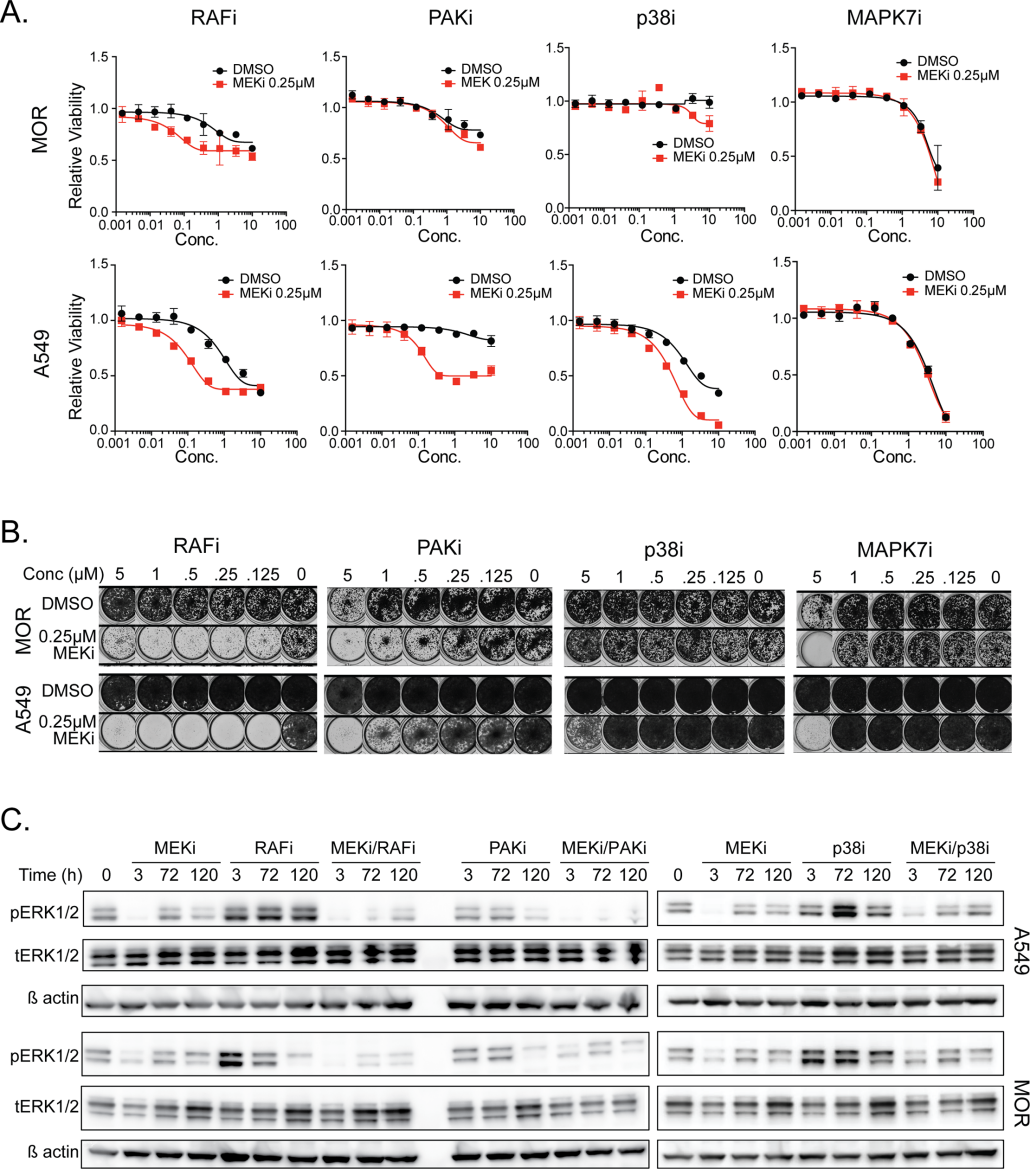
Inhibition of screen hits leads to reduced pathway rebound and is enhanced with longer term inhibition. **(A)** Dose response curves at 72 hrs in the presence of inhibitors to RAF, PAK1/2, p38 and MAPK7 +/- 0.25 μM MEK inhibitor. **(B)** 10 day clonogenic assay with dose response of RAF, PAK1/2, p38 and MAPK7 inhibitors +/- 0.25 μM MEK inhibitor. (**C)** Western blots showing changes in pERK1/2 levels with indicated inhibitors and time points indicated.

To further validate hits from the screen and test the utility of combination therapies, we utilized existing small molecule inhibitors against hits if they existed. As previously demonstrated [17, 18], the pan-RAF inhibitor, AZD-628, synergized with cobimetinib in cell viability assays using the A549, MOR, NCI-H2122 and NCI-H441 cell lines (Fig 3A and S3 Fig). Similarly, the PAK inhibitor G-5555 [19] and the p38 MAPK inhibitor BIRB-796 also showed combination benefit with cobimetinib. Surprisingly, the MAPK7 inhibitor, XMD17-109 [20], showed no ability to enhance the activity of cobimetinib in A549 or MOR cells in four-day cell viability assays (Fig 3A). As some combination effects may require longer treatment to elicit their effects, we extended the assay window to a 10-day clonogenic assay. Here again the combinations of pan-RAF and PAK inhibitors with cobimetinib showed clear combinatorial effects, similar to that seen in the shorter assay, however in this assay setting the effects of the p38 inhibitor were modest (Fig 3B). Interestingly, inhibition of MAPK7 resulted in a positive interaction with cobimetinib in 10-day clonogenic assays whereas this effect was absent in the short-term viability experiments (Fig 3B).

We hypothesized that the combination effects observed could be due to reduced reactivation of MAPK pathway signaling, particularly at later time-points. The pan-RAF and PAK inhibitors showed evidence for reduced pathway reactivation at later time points, suggesting a potential mechanism accounting for some of the combination effects of these compounds with cobimetinib (Fig 3C). In contrast, the p38 inhibitor did not affect the delayed reactivation of ERK phosphorylation, suggesting that any combination effects of this pathway are likely through different mechanisms.

### Inhibition of MAPK7 activity sensitizes some *KRAS* mutant lung lines to MEK inhibitor associated with reduced MAPK feedback activation

**Fig 4:**
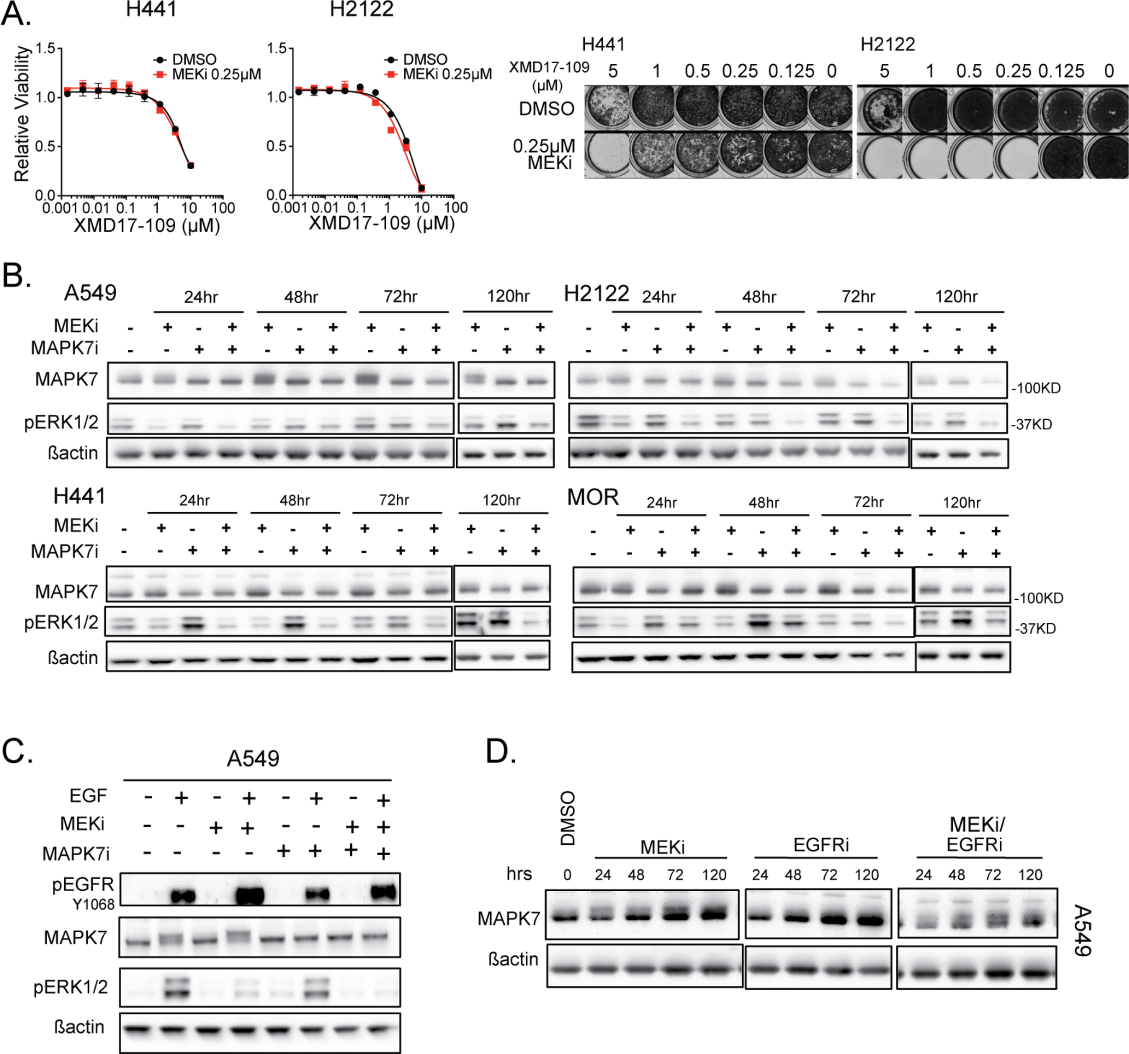
Inhibition of MAPK7 in combination with MEK inhibition leads to reduced MAPK pathway rebound and reduced viability. **(A)** Left: Dose response curves at 72 hrs for MAPK7 inhibitor +/- MEK inhibitor for NCI-H441 and NCI-H2122 cells. Right: 10 day clonogenic assay for the same combinations as the 72 hrs assay. **(B)** Western blot showing MAPK7 phosphorylation (band shift) and ERK1/2 phosphorylation in response to MEK inhibitor (0.25 μM), MAPK7 inhibitor (1 μM) and the combination at four time-points in A549, NCI-H2122, NCI-H441 and MOR cell lines. **(C)** Western blot showing phosphorylation changes in response to EGF addition (O/N starve, 10 min stimulation), MEK inhibitor (0.25 μM, 24 hrs), MAPK7 inhibitor (1 μM, 24 hrs) and combination, in the A549 cell line. **(D)** Western blot showing MAPK7 phosphorylation (band shift) in response to MEK inhibitor (0.25 μM), EGFR inhibitor (1 μM) and combination at four time points in the A549 cell line.

Cobimetinib and MAPK7 inhibitors showed slightly discrepant results depending on the assay and time points examined (Fig 3A and 3B), so we explored this in more detail using additional cell lines. Similar to A549 and MOR cells, H2122 and H441 cells also showed no additional inhibition of cell viability when cobimetinib was combined with XMD17-109, using a 4-day viability assay (Fig 4A). However, an 8-day clonogenic assay uncovered a more profound cooperative effect between these two inhibitors in these cell lines, especially in H2122 cells (Fig 4A). While inhibition of MAPK7 had little effect on RAS-MEK-ERK signaling activity on its own (in some cell lines it appeared to modestly increase ERK phosphorylation), it was effective at reducing pathway rebound at later time points in all cell lines tested (Fig 4B). MEK inhibition also increased MAPK7 mobility in some cell lines (most notably A549), which has previously been shown to be due to MAPK7 autophosphorylation [21] or an effect induced upon EGF treatment [22]. We hypothesized that MEK inhibition causes increased MAPK7 phosphorylation through the relief of negative feedback from ERK to EGFR and that this effect could be phenocopied by EGFR activation [23]. We confirmed that EGF treatment results in MAPK7 phosphorylation, and that MEK inhibition enhanced this effect (Fig 4C). However, EGFR inhibition did not prevent cobimetinib-induced MAPK7 phosphorylation, suggesting that inhibition of MEK causes MAPK7 phosphorylation through an EGFR-independent mechanism (Fig 4D).

### Genetic manipulation of MAPK7 cooperates with MEK inhibition in long-term viability and anchorage-independent growth assays

**Fig 5:**
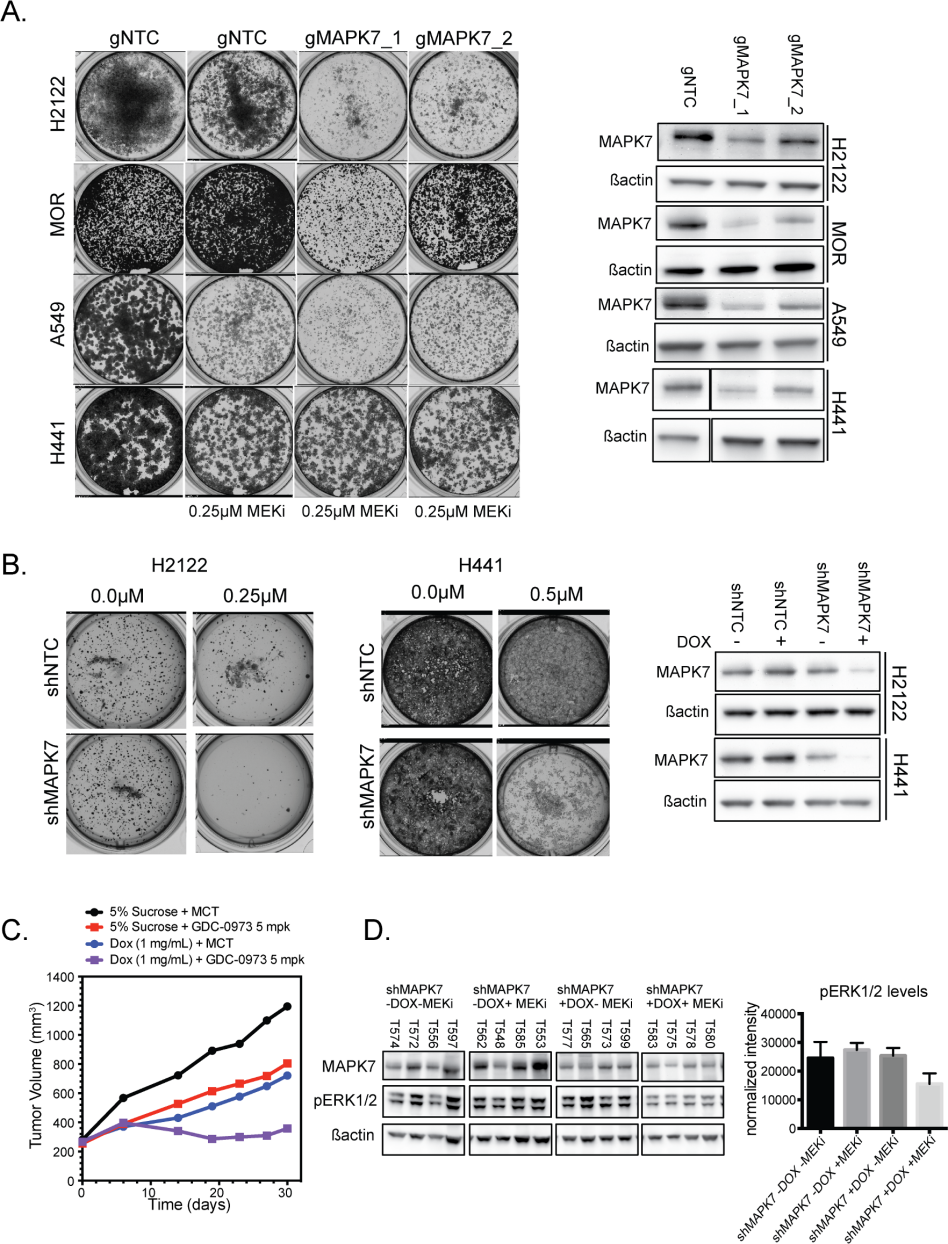
Loss of MAPK7 in combination with MEK inhibition leads to reduced viability in vitro and in vivo. **(A**) Left: Long-term growth assay showing the effect on cell proliferation at day 10 in NCI-H2122, MOR, A549, and NCI-H441 with MEK inhibitor alone and in combination with a control gRNA sequence and two sequences targeting *MAPK7*. Right: Western blot showing the level of MAPK7 protein loss observed with the labeled gRNA sequence at day 10 for each cell line. **(B)** Left: Long-term growth assay showing the effect on cell proliferation at day 10 in NCI-H2122 and NCI-H441 with MEK inhibitor alone and in combination with inducible shRNA mediated knockdown of *MAPK7*. Right: Western blot showing the level of MAPK7 protein loss observed with the indicated shRNA sequence at day 10 for both cell lines. **(C)** Plot showing NCI-H2122 shMAPK7 xenograft tumor volumes for tumors treated with vehicle or treated with MEK inhibitor, and in the presence or absence of doxycycline (Dox) induction of shRNA mediated knockdown. Tumor volumes are summarized using a mixed linear effects model. **(D)** Western blot showing levels and phosphorylation of MAPK7 and phosphorylation of ERK1/2 in four tumor samples from each arm at the end of the study. Right: Bar plot showing quantitation of ßactin normalized levels of pERK1/2 from the tumors shown in the western blot in the center.

While XMD17-109 was generated to target MAPK7, and shows little activity against 241 additional protein kinases [20], it was possible that the effects observed were due to off-target consequences, as seen in other studies [24]. Therefore, we complemented these inhibitor data using additional CRISPR- and RNAi-mediated reagents. Two independent gRNAs targeting *MAPK7* were effective at enhancing the consequences of MEK inhibition in clonogenic cell proliferation, in NCI-H2122, MOR, A549 and NCI-H441 cells (Fig 5A), whereas MAPK7 knockout alone had no effect on cell viability (S4 Fig). Similarly, shRNA-mediated knockdown of *MAPK7* caused a greater reduction in clonogenic growth compared to MEK inhibition alone (Fig 5B). To assess the impact of MAPK knockdown on tumor growth, we next evaluated the combination of MEK inhibition with *MAPK7* knockdown in the NCI-H2122 xenograft model. Inhibition of MEK, by cobimetinib (at 5 mg/kg, daily), or induction of shRNAs against *MAPK7* resulted in tumor growth inhibition, however the combination resulted in improved tumor growth inhibition, demonstrating in vivo validation of our in vitro findings (Fig 5C; shRNAs against NTCs as well as individual tumor plots shown in S5 Fig). The combination of MEK inhibition with MAPK7 knockdown resulted in improved suppression of ERK phosphorylation (Fig 5D).

## Discussion

CRISPR screens to identify targets for drug sensitization represent a powerful technique to evaluate appropriate combination strategies to treat indications currently underserved by existing therapeutic approaches, such as *KRAS* mutant NSCLC. Tumors harboring *KRAS* mutations are resistant to EGFR-directed therapies [25], and in some trials (but not all), showed greater resistance to standard chemotherapy treatment [26]. As MEK is activated in tumors expressing *KRAS* mutations, MEK inhibitors are a logical treatment option to explore in these patients. Unfortunately, MEK inhibitors have not shown any activity in this setting, either alone or in combination with EGFR inhibitors or chemotherapy [27–29]. As phosphoinositide 3-kinase (PI3K) is also activated by RAS, combinations of MEK and PI3K inhibitors had been proposed as a strategy to block RAS signaling with promise shown for the concept in preclinical models [30]. Unfortunately, these combinations have also been associated with significant toxicity in clinical trials, thereby limiting their benefit [31].

More recently, unbiased screening strategies have been employed to identify proteins and pathways to target in combination with MEK. An shRNA screening strategy identified *BCL2* as a gene that combined effectively with the MEK inhibitor selumetinib when depleted, and the BCL-2/BCL-XL inhibitor ABT-263 (navitoclax) shows strong synergy with MEK inhibitors in preclinical studies [32, 33]. In addition, an shRNA library screen against human kinases recently identified *FGFR1* as a strong sensitizing hit using trametinib, and FGFR inhibitors combined with trametinib were effective in *KRAS* mutant NSCLC xenografts as well as genetically engineered mouse models [15]. *FGFR1* was not identified in our CRISPR screen, probably because *FGFR1* is expressed at very low levels in MOR cells [34].

The screen described here identified components of the RAS/MAPK pathway itself as among the strongest hits when combined with MEK inhibition in mutant *KRAS* NSCLC cells, including *KRAS*, *RAF1* (encoding CRAF) and *MAPK1* (encoding ERK2). *KRAS* and *RAF1* were also previously identified in an shRNA screen using the MEK inhibitor PD0325901 [16], and pan-RAF inhibitors in combination with MEK inhibitors show promising efficacy in preclinical studies [18, 35]. We focused our attention on MAPK7 as this protein kinase is relatively poorly understood. While *MAPK7* was one of the strongest hits from the CRISPR screen, the gene encoding the direct activator of MAPK7 activity, *MEK5*, was also identified as being significantly depleted (S1 Table), further supporting the role of this pathway in mediating resistance to MEK inhibition. In addition, *MAPK7* emerged as a consistent hit across the four validation cell lines, in contrast to some of the strong hits in the screening line MOR that were not depleted in additional cell lines (e.g. *CHUK*/*IKKα*).

**Fig 6:**
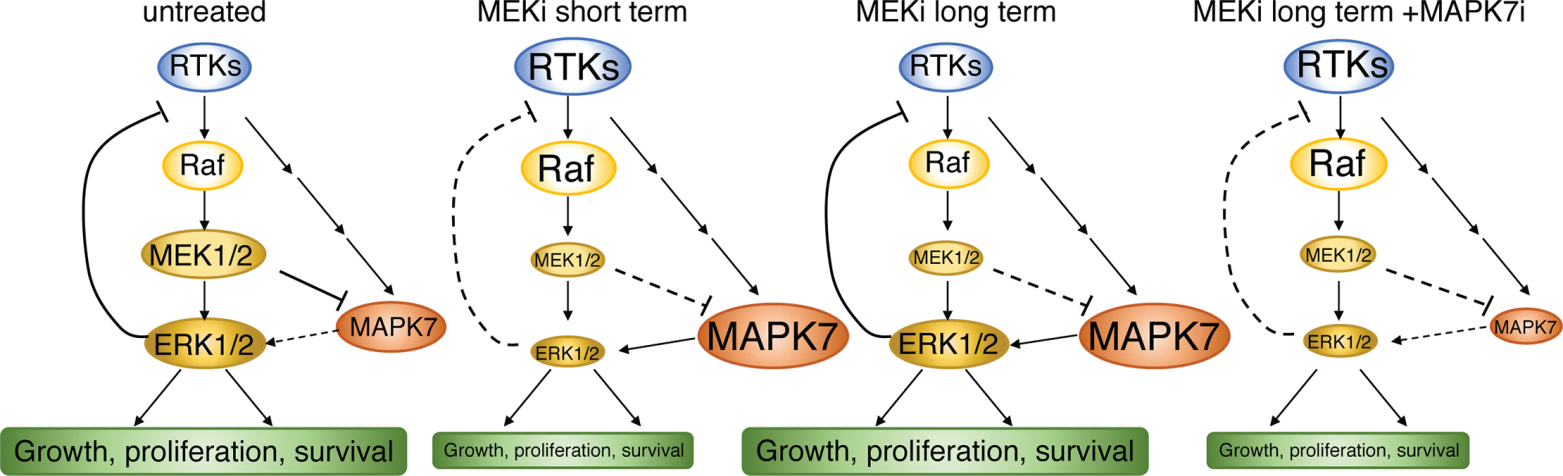
Model for the relationship between MEK1/2 inhibition and MAPK7 inhibition. In untreated proliferating cells all signaling components show basal activity and active ERK1/2 relay negative feedback signals upstream of Raf kinases. Following MEK inhibitor treatment, ERK activity is reduced, decreasing cell proliferation, but relieving negative feedback to RTKs/Raf and MAPK7. Eventually this increased MAPK7 activity can contribute to ERK1/2 reactivation even in the presence of MEK inhibitors. MAPK7 knockout/knockdown or inhibition prevents the delayed reactivation of ERK1/2 in the presence of MEK inhibitors thereby causing a more pronounced inhibition of cell proliferation, especially in long term assays.

MAPK7 shares 66% identity with ERK1/2 in the kinase domain, but has an extended C-terminus containing a nuclear localization signal (NLS) and a putative transcriptional activation domain. It is activated by growth factors as well as cellular stresses [36]. We have shown that MAPK7 phosphorylation increases in response to the MEK inhibition, consistent with a role in mediating MEK inhibitor resistance. Similar activation of MAPK7 in response to MEK inhibitor treatment has previously been shown [37–39], and such activation could represent a common mechanism of resistance for MEK inhibitor treatment. In our experiments, this potential co-dependency does not appear in short-term growth assays, consistent with other studies [40], but manifests in both long-term clonogenic assays, as well as tumor xenograft studies. It isn’t clear whether the therapeutic index of MEK/MAPK7 inhibitor combination will be similar or different to serial combination inhibition of the Raf/MEK/ERK pathway, which is highly effective at inhibiting tumor cell proliferation, but which is also somewhat toxic [41]. Addition of MAPK7 inhibitors to Raf/MEK or MEK/ERK inhibitor combinations would therefore be interesting to test, to determine whether the predicted benefit seen in experimental conditions (Fig 6), would be seen in clinical settings. Interestingly, the consequences of MAPK7 inhibition appear to extend beyond MEK inhibitors, and in fact potentiate the effects of multiple cytotoxic agents including etoposide, trastuzumab, fulvestrant, tamoxifen, docetaxel, doxorubicin, cisplatin, vinorelbine, imatinib, dexamethasone, bortezomib, cytarabine, crizotinib and 5-FU (reviewed in [42]). Our results therefore support MAPK7 representing a potentially tractable target for various tumor indications when combined with appropriate therapeutic agents.

## Supporting information

S1 Table Names and sources of inhibitor compounds used in this manuscript. (XLSX)

S2 Table Gene names and gRNA sequences used in the pooled CRISPR screen described in Fig 1. (XLSX)

S3 Table Gene names and gRNA sequences used in the CRISPR minipool described in Fig 2A. (XLSX)

S4 Table Gene names and gRNA sequences used in the arrayed CRISPR viability screen described in Fig 2B. (XLSX)

S1 Fig **(A)** Dose response of the MEK inhibitor cobimetinib and the ERK inhibitor GDC-0994 in the NSCLC cell line MOR. Viability was measured using CellTiter-Glo® reagents to measure cellular ATP levels after 4 day drug treatment. **(B)** MOR cells were treated with 1 μM GDC-0994 or 500 nM cobimetinib for 3, 24 or 48 hours. Cell lysates were then separated by SDS-PAGE and protein levels examined by Western blotting. **(C)** Schematic of the CRISPR screen design.
(TIF)

S2 Fig A549 cells were infected with lentiviruses expressing the indicated gRNAs. Three days following selection with puromycin, 50 nM cobimetinib or DMSO was added, and cell viability measured using CellTiter-Glo® after one, two, three or four passages.
(TIF)

S3 Fig NCI-H441 and NCI-H2122 cells were treated with increasing doses of RAF, PAK, p38 or MAPK7 inhibitors, in the presence or absence of 0.25 μM cobimetinib. Viability was measured after four days using CellTiter-Glo®.
(TIF)

S4 Fig The indicated cell lines were infected with lentiviruses expressing gRNAs targeting control (NTC) or two different MAPK sequences, then selected in puromycin for 10 days. Resulting colonies were stained using crystal violet.
(TIF)

S5 Fig **(A)** Plot showing NCI-H2122 shNTC xenograft tumor volumes for tumors treated with vehicle or treated with MEK inhibitor, and in the presence or absence of doxycycline (Dox). Tumor volumes are summarized using a mixed linear effects model. **(B)-(D)** Tumor growth curves from individual shMAPK7 mice treated with the indicated reagents.
(TIF)
