# Supplementary figures and images for: A CRISPR screen identifies MAPK7 as a target for combination with MEK inhibition in KRAS mutant NSCLC
Source: PLoS One. 2018 Jun 18;13(6):e0199264. doi: 10.1371/journal.pone.0199264 (PMC6005515; doi:10.1371/journal.pone.0199264)

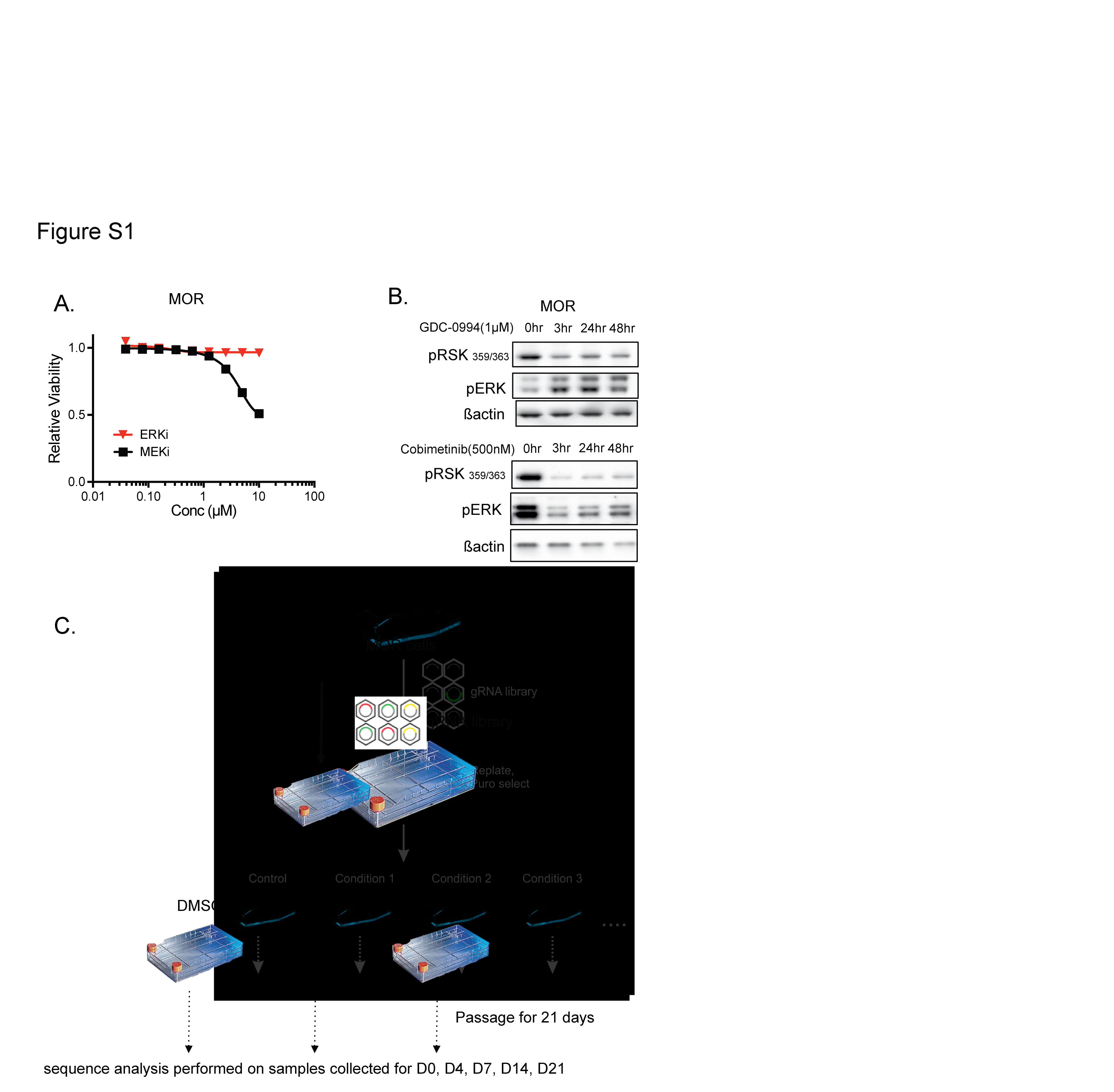

Supplement: S1 Fig — (A) Dose response of the MEK inhibitor cobimetinib and the ERK inhibitor GDC-0994 in the NSCLC cell line MOR. Viability was measured using CellTiter-Glo® reagents to measure cellular ATP levels after 4 day drug treatment. (B) MOR cells were treated with 1 μM GDC-0994 or 500 nM cobimetinib for 3, 24 or 48 hours. Cell lysates were then separated by SDS-PAGE and protein levels examined by Western blotting. (C) Schematic of the CRISPR screen design. (TIF) [file pone.0199264.s005.tif]

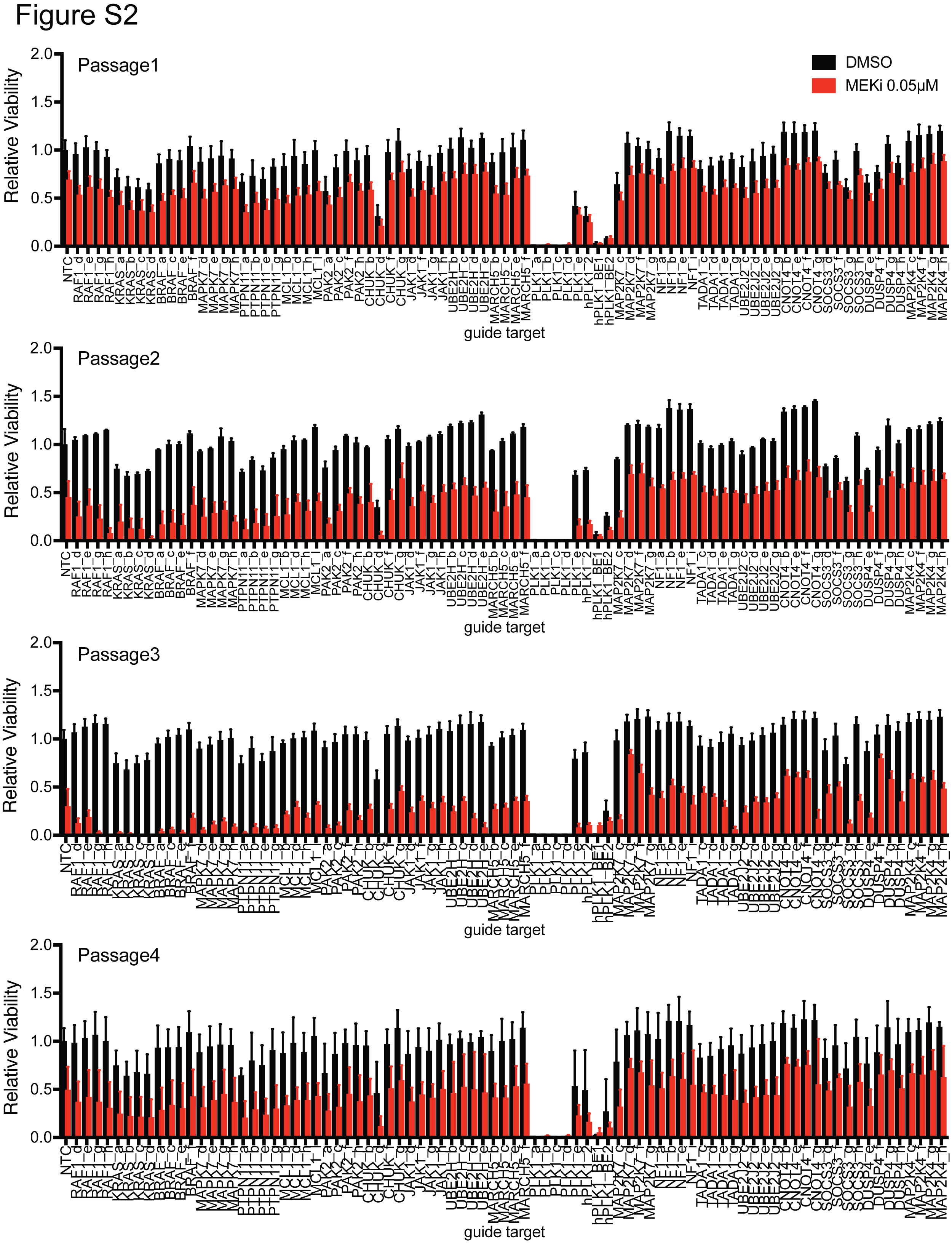

Supplement: S2 Fig — Three days following selection with puromycin, 50 nM cobimetinib or DMSO was added, and cell viability measured using CellTiter-Glo® after one, two, three or four passages. (TIF) [file pone.0199264.s006.tif]

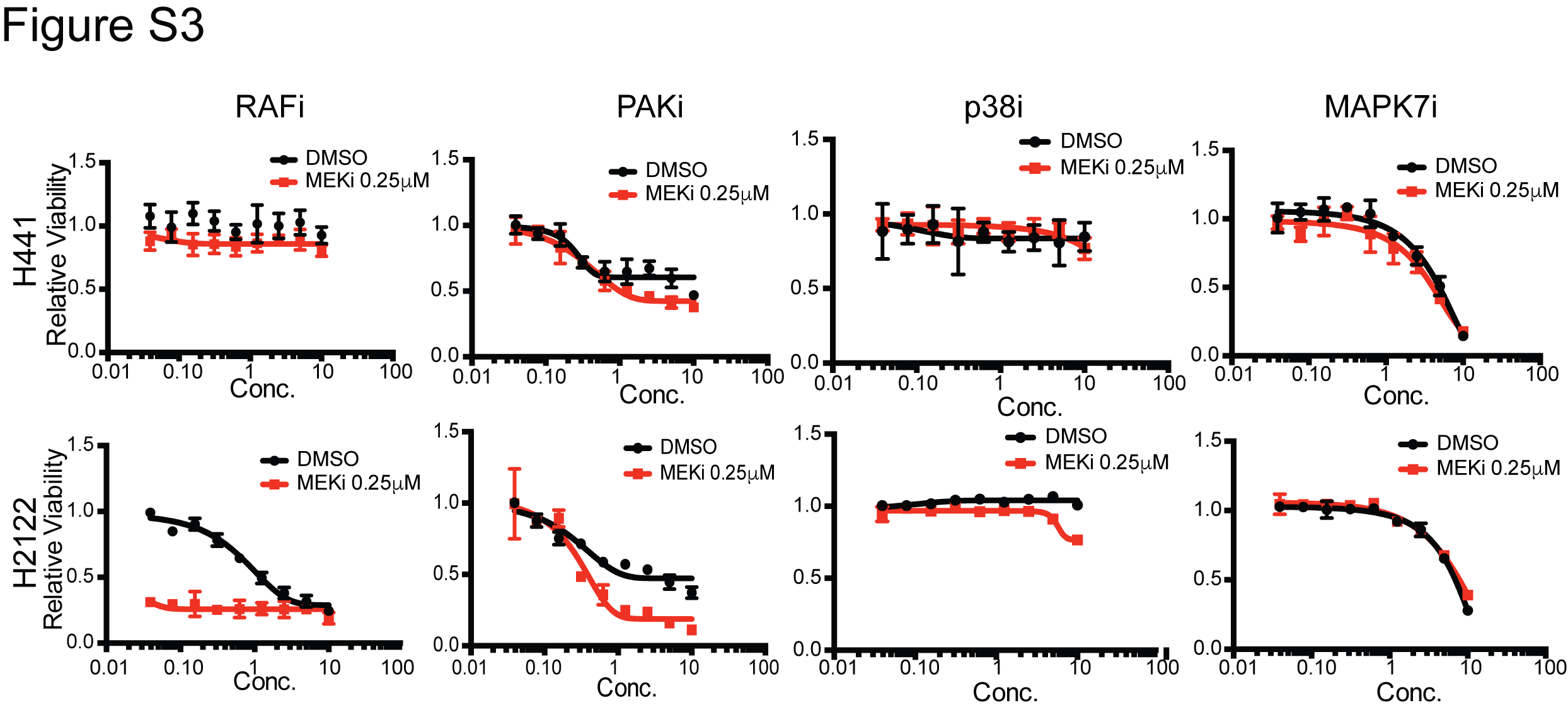

Supplement: S3 Fig — Viability was measured after four days using CellTiter-Glo®. (TIF) [file pone.0199264.s007.tif]

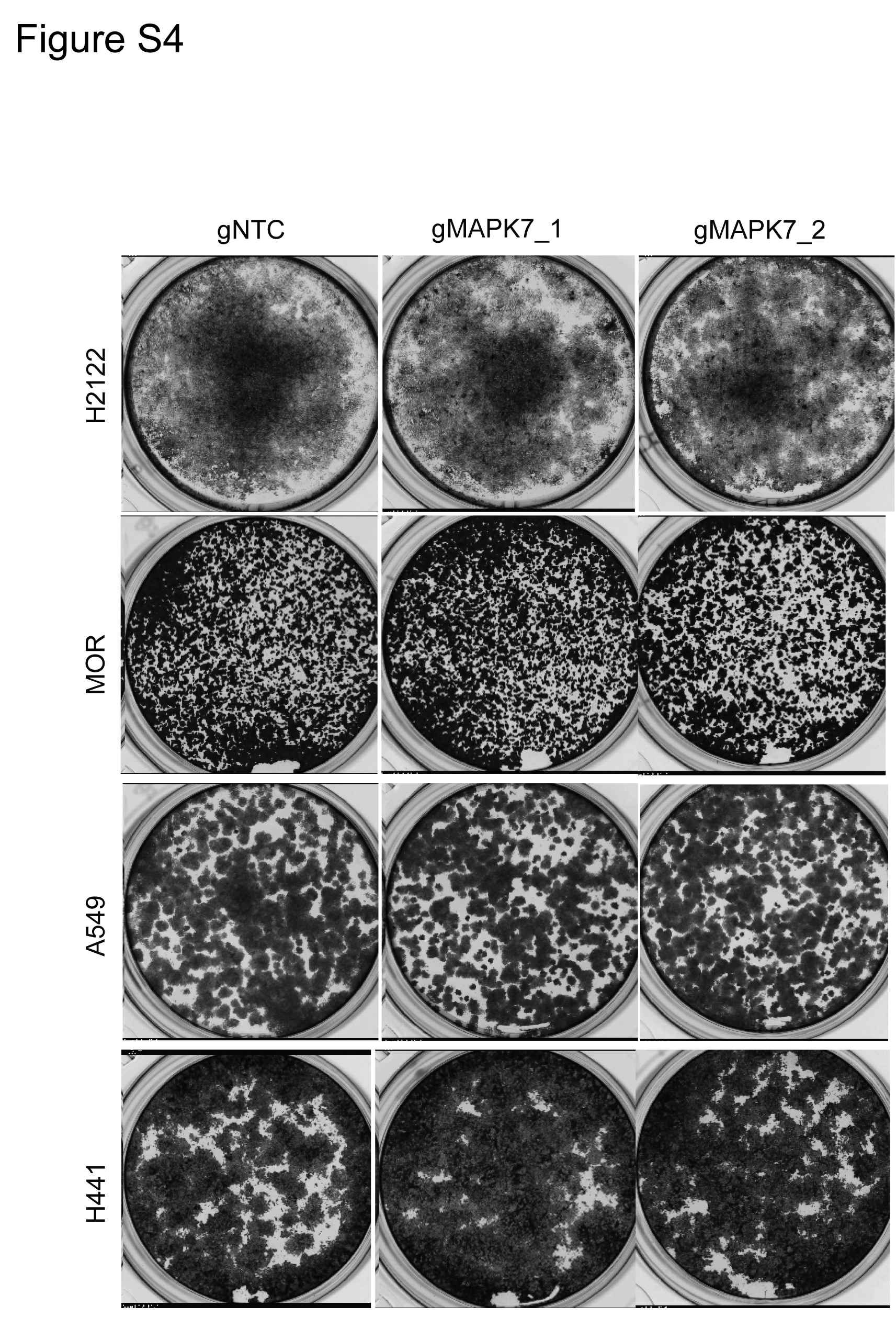

Supplement: S4 Fig — Resulting colonies were stained using crystal violet. (TIF) [file pone.0199264.s008.tif]

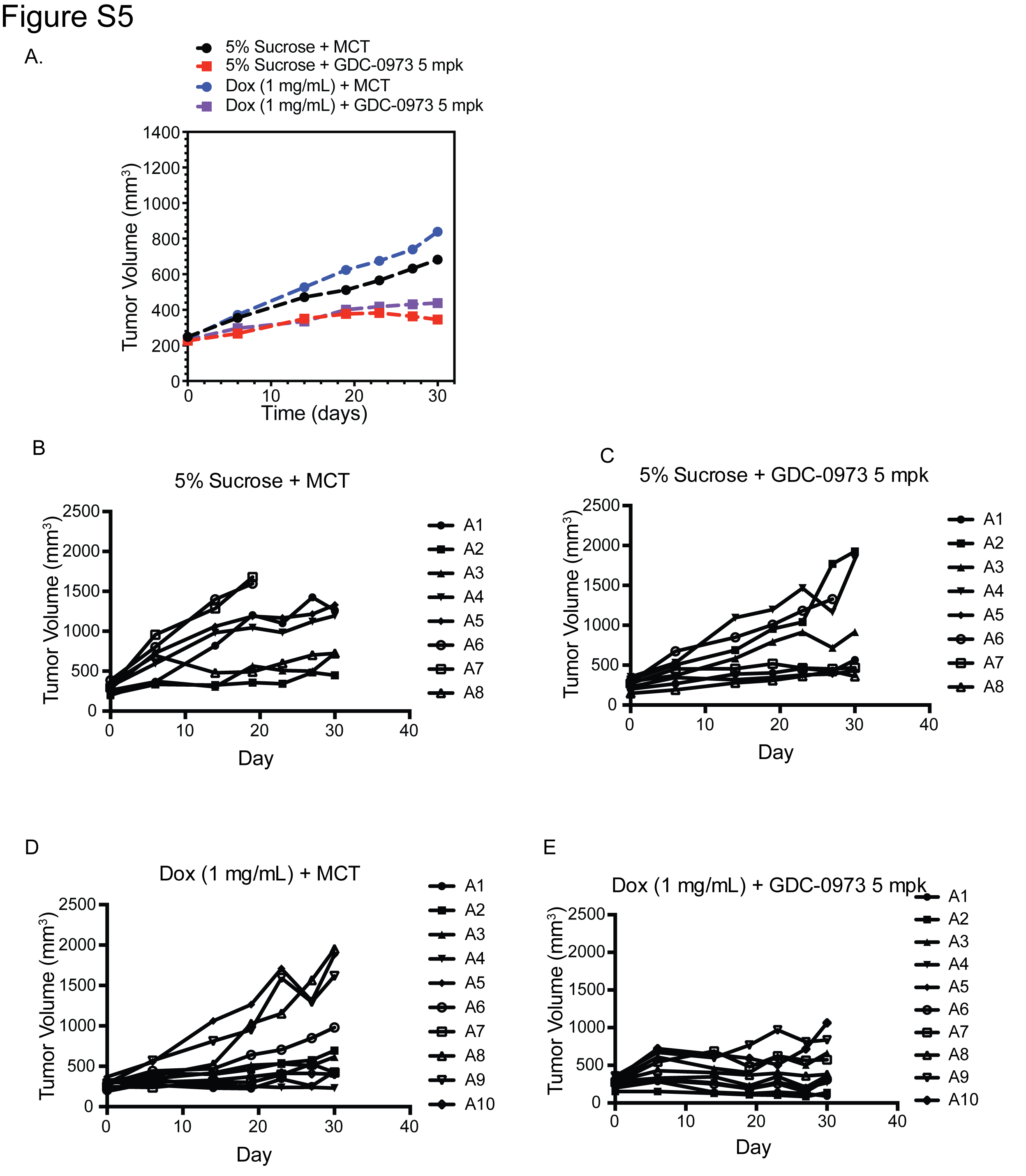

Supplement: S5 Fig — (A) Plot showing NCI-H2122 shNTC xenograft tumor volumes for tumors treated with vehicle or treated with MEK inhibitor, and in the presence or absence of doxycycline (Dox). Tumor volumes are summarized using a mixed linear effects model. (B)-(D) Tumor growth curves from individual shMAPK7 mice treated with the indicated reagents. (TIF) [file pone.0199264.s009.tif]
